# Feasibility study of using Stereotactic Field Diode for field output factors measurement and evaluating three new detectors for small field relative dosimetry of 6 and 10 MV photon beams

**DOI:** 10.1002/acm2.13007

**Published:** 2020-10-19

**Authors:** Attia Gul, Shigekazu Fukuda, Hideyuki Mizuno, Nakaji Taku, M. Basim Kakakhel, Sikander M. Mirza

**Affiliations:** ^1^ QST Hospital National Institutes for Quantum and Radiological Science and Technology Chiba Japan; ^2^ Department of Physics & Applied Mathematics Pakistan Institute of Engineering and Applied Sciences (PIEAS) Islamabad Pakistan

**Keywords:** correction factors, field output factors, PTW31023, RAZOR diode, RAZOR nano chamber, small field dosimetry, stereotactic field diode

## Abstract

This study assesses the feasibility of using stereotactic field diode (SFD) as an alternate to gaf chromic films for field output factor (FF) measurement and further evaluating three new detectors for small field dosimetry. Varian 21EX linear accelerator was used to generate 6 and 10 MV beams of nominal square fields ranging from 0.5 × 0.5 cm^2^ to 10 × 10 cm^2^. One passive (EBT3 films) and five active detectors including IBA RAZOR diode(RD), SFD, RAZOR nanochamber (RNC), pinpoint chamber (PTW31023), and semiflex chamber (PTW31010) were employed. FFs were measured using films and SFD while beam profiles and percentage depth dose (PDD) distribution were acquired with active detectors. Polarity (*k_pol_*) and recombination (*k_s_*) effects of ion chambers were determined and corrected for output ratio measurement. Correction factors (CF) of RD, RNC, and PTW31023 in axial and radial orientation were also measured. Stereotactic field diode measured FFs have shown good agreement with films (with difference of <1%). RD and RNC measured beam profiles were within 3% deviation from the SFD values. Variation in *k_pol_* with field size for RNC and PTW31023 was up to 4% and 0.4% (for fields ≥ 1 × 1 cm^2^), respectively, while variation in *k_s_* of PTW31023 was <0.2 %. The maximum values of CF have been calculated to be 5.2%, 2.0%, 13.6%, and 25.5% for RD, RNC, PTW31023‐axial, and PTW31023‐radial respectively. This study concludes that SFD with appropriate CFs as given in TRS 483 may be used for measuring FFs as an alternate to EBT3 films. Whereas RD and RNC may be used for beam profile and PDD measurement in small fields. Considering the limit of usability of 2%, RNC may be used without CF for FF measurement in the smallfields investigated in this study.

## 1 INTRODUCTION

In recent years, the use of small fields (<4 × 4 cm^2^) in radiation therapy has increased many folds after the introduction of modern radiotherapy techniques such as intensity modulated radio therapy (IMRT), stereotactic radiosurgery (SRS), stereotactic body radiotherapy (SBRT) etc. Dose measurement in small fields has been a challenge due to various problems such as lack of charged particle equilibrium, source occlusion and penumbra overlapping.^1^, ^2^, ^3^ This is further complicated by the perturbations from different components of the detector, as well as the size of the detector comparable or larger than the radiation field.^4^, ^5^, ^6^ The last decade has witnessed a lot of effort for identification of suitable detectors and/ or applying appropriate corrections to employ these detectors for accurate dose measurement in small fields.^7^, ^8^, ^9^, ^10^, ^11^ Yet, there is no one‐size‐fits‐all solution available for small field dosimetry with each detector having its own pros and cons.^12^, ^13^ However, new detectors are being introduced to meet the above‐mentioned challenges.

For field output factor (FF) measurement, volume averaging effect (due to the detector size) and fluence perturbation (due to the existence of high‐density material in any detector) are the major detector related challenges. Therefore, output correction factors (CF) are required to correct detector response in small fields for the determination of FFs.^14^ A detector is recommended for FF measurement with appropriate corrections if its CF is within ±5% for that particular field size.^13^ While some studies have suggested that it may be used without any correction if its response is within ±2% of the actual FF.^15^, ^16^, ^17^

GAFCHROMIC EBT films due to their very high spatial resolution, weak energy dependence and water‐equivalence (z_eff_ = 7.26), have been recommended for use as gold standard for accurate FF measurement. Hence, films can be employed for CF determination of other large volume and high density detectors using detector to detector correction approach.^13^, ^18^ However, tedious processing, nonreusability and passive nature of films require exploring other active detectors suitable for use as a reference. In this regard, unshielded diodes, like stereotactic field diode (SFD), could be a good choice due to their reduced perturbation and smaller volume, thus resulting in smaller CFs as reported in literature compared to shielded diodes and small vented air ion chambers (IC).

IAEA and AAPM have jointly published a code of practice (CoP TRS 483) for dosimetry of static small fields.^13^ It provides guidelines for the selection of a detector for reference and relative small field dosimetry along with CFs of several detectors to measure FFs. Many small field detectors have become available after publication of this CoP, including IBA RAZOR diode (RD), RAZOR nanochamber (RNC), and PinPoint chamber (PTW31023). For RD detectors some published data are available for CF measurement and beam profiling in small fields. For instance, Cagni et al have measured the data for RD in 10 MV flattening filter free (FFF) beam for circular collimators of 6 to 50 mm diameter.^19^ Whereas, Reggiori et al have emphasized primarily on the characterization of the detector under FFF beam and not on the determination of its CFs.^20^ Liu et al have determined the CFs in the circular fields range of 5 to 30 mm, employing 30 mm as reference field.^21^ Still these published results need to be validated for different beam lines and collimation systems. To the best of our knowledge novel PTW31023 with a new design has not been yet investigated for beam profile and depth dose measurement in small fields. However, It has been characterized for reference and relative dosimetry in the conventional fields by Bushing et al and Bruggmoser et al.^22^, ^23^ Recently, it has also been studied by Casar et al for determination of CFs in radial and axial orientations.^24^ However, the response has not been corrected for polarity and recombination effects as recommended by TRS483. Similarly, RNC, the smallest commercially available IC, has been characterized for different dosimetric parameters specially the polarity and stem effects.^25^, ^26^, ^27^ A couple of authors have reported output ratio measurements using RNC as well.^28^, ^29^, ^30^ Casar et al has also determined the CFs for this detector in radial and axial directions without correcting its response for polarity effect.^24^ However, before deploying these detectors for small field relative dosimetry further assessment is needed. Furthermore, the measurement setup is not uniform, as per recommendation of TRS 483, in most of the above‐mentioned studies. Therefore, it is important to explore the measurement of CFs of these detectors following TRS 483 protocol, while considering polarity and recombination effects.

The present study aims at investigating the application of SFD as reference detector and evaluating three new detectors (RD, RNC, and PTW31023) not included in TRS483, for relative dosimetry of small fields under 6 and 10 MV beams collimated with jaws and multi leaf collimators (MLCs). The CFs of RD, RNC, and PTW31023 would be a valuable addition to the TRS 483 published data. Specific objectives of the study are as follows:

- To evaluate the SFD with its corresponding CFs (published in TRS 483), as a substitute of EBT3 films, for determination of FFs of small fields.
- To investigate RD, RNC, and PTW31023 for measuring beam profiles and depth dose distribution in small fields.
- To investigate the effect of polarity and ion recombination for RNC and PTW31023 ICs in small fields.
- To determine the CFs of RD, RNC, and PTW31023 and to investigate the field size limit for each detector for correction less FF measurements in small fields.

## 2 **MATERIALS AND METHOD**S

### 2.A Beam line and detectors

**Fig. 1:**
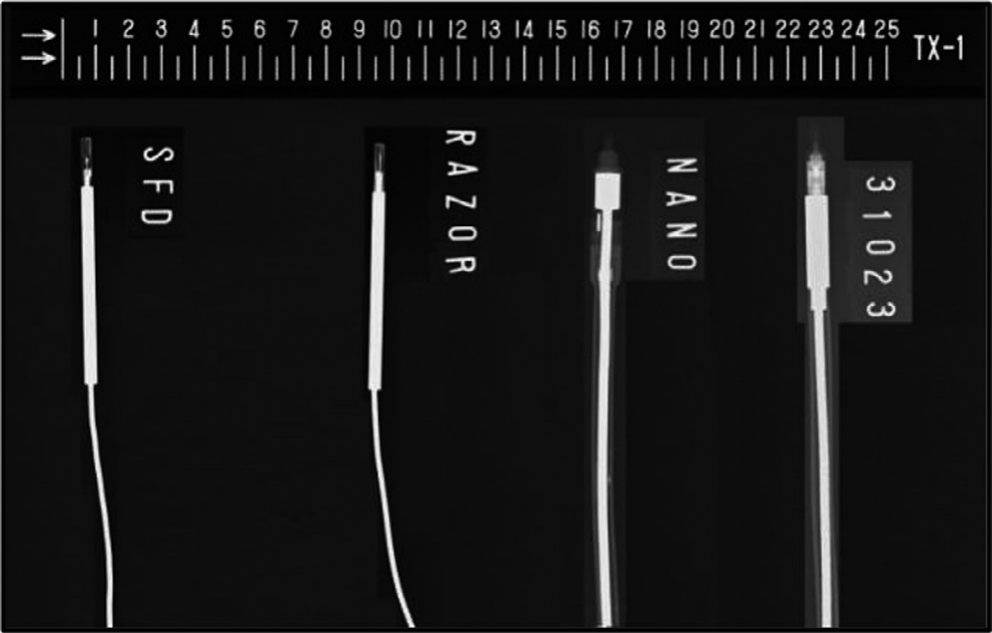
Radiograph of small field detectors used in this work (left to right) stereotactic field diode, RAZOR Diode, RAZOR Nanochamber, and PTW31023. The scale above is graduated in cm.

In this study, VARIAN CLINAC 21EX was used to generate 6 and 10 MV photon beams with a dose rate of 300 MU/min. The reference output of linear accelerator was 1 cGy/MU at the depth of maximum dose (d_max_) as measured employing the IAEA standard protocol TRS398 for absorbed dose determination.^31^ One passive detector, that is, GAFCHROMIC EBT3 films (ASHLAND) and five active detectors were employed. Active detectors included two diodes:Scanditronix IBA stereotactic field diode (SFD) and IBA RAZOR diode (RD) and three ionization chambers (IC) comprising IBA RAZOR nanochamber (RNC), pinpoint chamber PTW31023 and semiflex chamber PTW31010. Specifications of these detectors are presented in Table 1, while their radiographs are shown in Fig. 1.

**Table 1:** Technical specifications of the detectors used in this work.

Detectors	Active material/additional components	Shape of active material	Dimensions of active material
Stereotactic field diode (SFD)	p‐silicon	Disk	Diameter = 0.6 mm Thickness = 0.06 mm Volume = 0.017 mm^3^
RAZOR diode (RD)	p‐silicon	Disk	Diameter = 0.6 mm Thickness = 0.02 mm Volume = 0.006 mm^3^
RAZOR nanochamber (RNC)	Air cavity Central electrode = Graphite Wall = Shonka (C‐552)	Spherical	Diameter = 2 mm Volume = 3 mm^3^
PTW 31023 pinpoint chamber (PTW31023)	Air Cavity Central electrode = Aluminum Wall = PMMA with inner layer of conductive graphite	Cylindrical	Diameter = 2 mm Length = 5 mm Volume = 15.7 mm^3^
PTW 31010 semiflex chamber	Air cavity Central electrode = Aluminum Wall = PMMA with inner layer of conductive graphite	Cylindrical	Diameter = 5.5 mm Length = 6.5 mm Volume = 0.125 cm^3^
GAFCHROMIC EBT3 films	Active layer consisting of H, C, O, Al and Li sandwiched between two polyester substrates	2D	0.809 × 10^−3^ mm^a^

^a^ Active volume of EBT3 films is v = d^2 ^× t, where d = 0.17 mm that is, pixel size for 150 dpi resolution and t = 28 µm that is, thickness of active layer.

SFD and RD are unshielded diodes that have demonstrated lesser perturbation for small fields. Both RNC and PTW31023 have the same cavity radius, that is, 1 mm, but the active volume of PTW31023 is larger due to its cylindrical shape unlike RNC which has spherical air cavity. RNC is the smallest commercially available IC. Novel PTW31023 IC is the successor of its old version PTW31014 with improved guard ring and inner electrode design to make it a reference class detector.

### 2.B Measurements

**Fig. 2:**
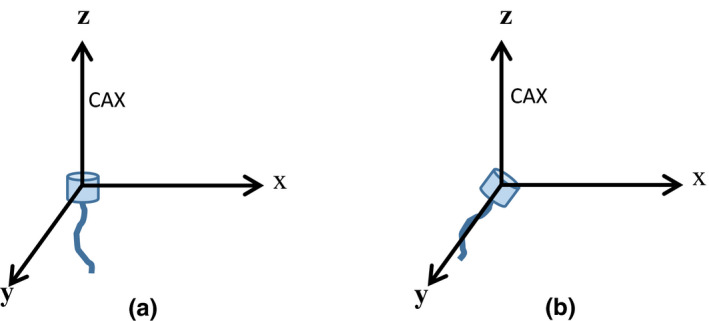
Orientation of detectors in water phantom (a). Axial and (b). Radial. CAX is the beam's central axis.

Small field data were acquired for 6 and 10 MV photon beams with nominal square field sizes of 0.5 × 0.5 cm^2^, 1 × 1 cm^2^, 1.5 × 1.5 cm^2^, 2 × 2 cm^2^, 2.5 × 2.5 cm^2^, 3 × 3 cm^2^, 4 × 4 cm^2^, and 10 × 10 cm^2^ collimated with jaws and MLCs (i.e. 32 combinations of field size, energy and collimation system). Two collimation systems were used to increase the range of effective field size. For jaw defined fields, MLCs were parked at fully open position. Whereas for all MLC shaped fields, jaws were fixed at 10 × 10 cm^2^ field size. To remain consistent with TRS 483 nomenclature, the terms machine specific reference (msr) field and clinical field would be used in this work for field size of 10 × 10 cm^2^ and the rest of the fields respectively. All measurements were carried out in an IBA blue water phantom with iso‐centric setup, that is, 10 cm depth with source to surface distance (SSD) of 90 cm. IBA scanner and electrometer RFA300 were employed for acquisition of dose distribution data with positional accuracy of 0.3 mm whereas FLUKE electrometer Model 35040 was used to measure charge for calculation of output ratios. Diode detectors were aligned in water phantom with axial orientation [Fig. 2(a)] whereas ion chambers were placed with radial direction [Fig. 2(b)]. Additional measurements for PTW31023 were carried out in axial direction to investigate the effect of orientation. All these orientations are in accordance with the TRS 483 recommendations. Before measuring the FFs and output ratio (OR), the detector's position in the center of radiation field was verified from inline and crossline profiles at the depth of measurement for the smallest (0.5 × 0.5 cm^2^) and largest (10 × 10 cm^2^) nominal fields used in this study. For each field size, detector's signal (*M*) per 100 monitor units (MUs) was measured at least five times and then averaged.

#### 2.B.1 EBT3 film processing

A strict protocol was followed during the whole process of film dosimetry from cutting to scanning and the analysis to reduce the uncertainty in dose measurement. Films were cut into pieces of 3 × 3 cm^2^ at least 24 h before irradiation and each piece was marked to ensure consistency in orientation during irradiation and scanning. These pieces were placed between the slabs of solid water phantom (Gammex RMI^®^) perpendicular to the beam central axis (CAX) at the depth of 10 cm with SSD = 90 cm. For calibration curve, films were irradiated with known doses of up to 5 Gy under both 6 and 10 MV beams in msr field to expose them with homogenous dose. For FF measurement at least three films (total of 96 films for 32 combinations of field size, energy and collimation type) were irradiated for number of MUs calculated to deliver the total dose of 3.5 Gy for each combination of field size and energy using SFD corrected FFs as explained further in Section 2.B.3. Both calibration and FF films were scanned 24 h after exposure with EPSON ES‐10000G (Seiko Epson Corporation) flatbed scanner and images were acquired with Film Scan ver. 4.0.0 software with scan parameters of 150 dpi resolution, three scans per film (taking average for dose calculation), 48‐bit RGB mode and red color channel. Film analysis was carried out with DD System ver. 14.65 software(R‐Tech. Inc. Japan)and central dose was measured in square region of interest (ROI) with size of 0.5 × 0.5 mm^2^ drawn in the center of each exposed film employing ROI Analysis routine. The final dose was measured as the average of three films irradiated for each combination of field size, energy, and collimation system. The uncertainty in terms of relative standard deviation (1 rel. SD) was also calculated.

#### 2.B.2 Effective field size

Effective field size (*s_eff_*) against each nominal field collimated with jaws and MLCs was measured with SFD using the following formula:

(1) $$s_{\mathrm{eff}}=\sqrt{x\times y}$$

where x and y are full width half maxima (FWHM) of crossline and inline beam profiles, respectively, measured at 10 cm depth in water phantom at SSD = 90 cm.

#### 2.B.3 Field output factors (FF)

FFs were measured with SFD and EBT3 films as described under.

- **SFD**

For SFD, the following two methods were employed to measure the FF.

- **Direct method**

FFs were determined with direct method for all fields using the formula given as under.

(2) $$FF_{f_{\mathrm{clin}}}=\frac{M_{f_{\mathrm{clin}},SFD}}{M_{f_{\mathrm{msr}},SFD}}\times CF_{f_{\mathrm{clin}},SFD}$$

where $M_{f_{\mathrm{clin}},SFD}$ and $M_{f_{\mathrm{msr}},SFD}$ are the detector's readings (average of five readings) for clinical and msr fields, respectively, and $CF_{f_{\mathrm{clin}},SFD}$ is the correction factor of SFD given in TRS 483. Linear interpolation method was used to calculate the CFs of those fields for which CFs are not provided in TRS 483. For instance, CF of 6 MV effective field size of 0.56 cm (against nominal field of 0.5 × 0.5 cm^2^ defined with MLCs) was calculated from the linear interpolation of CFs given for 0.5 and 0.6 cm in TRS 483.

- **Intermediate field method (IFM)**

Field output factors with SFD were also calculated employing IFM as given in Eq. (3). Field size of 3 × 3 cm^2^ (the smallest field for which lateral charged particle equilibrium holds for both energies) was chosen as intermediate field^32^ whereas semiflex PTW31010 (mentioned as subscript semi) ion chamber was used to limit the effect of energy dependence of SFD.

(3) $$FF_{f_{\mathrm{clin}}}=\left[\frac{M_{f_{\mathrm{clin}},SFD}}{M_{f_{\mathrm{int}},SFD}}\times CF_{f_{\mathrm{clin}}\leftarrow f_{\mathrm{int}},SFD}\right]\times \left[\frac{M_{f_{\mathrm{int}},semi}}{M_{f_{\mathrm{msr}},semi}}\times CF_{f_{\mathrm{int}}\leftarrow f_{\mathrm{msr}},semi}\right]$$

where $CF_{f_{\mathrm{int}}\leftarrow f_{\mathrm{msr}},semi}$ is the CF of semiflex chamber for intermediate field with reference to msr field while $CF_{f_{\mathrm{clin}}\leftarrow f_{\mathrm{int}},SFD}$ is the CF of SFD for clinical field with reference to intermediate field and can be calculated as follows:

(4) $$CF_{f_{\mathrm{clin}}\leftarrow f_{\mathrm{int}},SFD}=\frac{CF_{f_{\mathrm{clin}},SFD}}{CF_{f_{\mathrm{int}},SFD}}$$

The correction factors for SFD and semiflex IC in above stated formulae were taken from TRS 483. Linear interpolation method was used to find CFs where needed as explained above.

- **EBT3 films**

Field output factors with EBT3 films were calculated employing method proposed by Garnier et al^33^ as described in Eq. (5). In this method, films were irradiated for number of MUs that were calculated to obtain an absolute dose of 3.5 Gy for each field size of both energies collimated with jaws and MLCs. Number of MUs for all fields were calculated from SFD measured FFs [Eq. (2)] and given in Table 2. Advantages of this method include the irradiation of films in an ideal dose range and getting the same signal to noise ratio and hence the same uncertainty for all field sizes that would be compromised for smaller fields otherwise.^33^ Field output factors with EBT films were calculated using Eq. (5).

(5) $$FF_{f_{\mathrm{clin}}}=\frac{D_{f_{\mathrm{clin}},film}}{D_{f_{\mathrm{msr}},film}}\times \frac{MU_{f_{\mathrm{msr}}}}{MU_{f_{\mathrm{clin}}}}$$

where $D_{f_{\mathrm{clin}},film}$ and $D_{f_{\mathrm{msr}},film}$ are the absorbed dose measured with films while $MU_{f_{\mathrm{clin}}}$ and $MU_{f_{\mathrm{msr}}}$ are the number of MUs for which films were irradiated in clinical and msr fields respectively.

**Table 2:** Number of MUs delivered for film irradiation under different field sizes. MUs are calculated from SFD measured FFs to deliver dose of 3.5 Gy.

Nominal field size (cm^2^)		0.5 × 0.5	1 × 1	1.5 × 1.5	2 × 2	2.5 × 2.5	3 × 3	4 × 4	10 × 10
6 MV	Jaws	896	660	601	575	560	545	524	450
	MLCs	812	634	587	555	542	524	504	450
10 MV	Jaws	998	649	561	522	500	486	467	412
	MLCs	861	613	545	502	484	465	447	412

#### 2.B.4 **Beam profiles and percent depth dose (PDD**)

Crossline beam profiles and PDDs were acquired in small fields collimated with Jaws with four active detectors (SFD, RD, RNC, and PTW31023) in water phantom using scanning resolution of 0.2 and 0.5 mm respectively. For crossline profiling, ICs were placed in inline orientation [as shown in Fig. 2(b)] to reduce the stem effect.^27^ FWHM and penumbra (distance between 80% and 20% dose) of beam profiles as measured with different detectors were also compared.

Beam profiles with all detectors were measured several times (two to five) on at least two different days to estimate the uncertainty (1SD) that has been found to be <0.19 and 0.17 mm for FWHM and penumbra values respectively.

#### 2.B.5 Polarity and recombination correction factors of ion chambers

Small vented ICs exhibit a significant polarity and ion recombination effect that may vary with field size. Polarity correction factor (*k_pol_*) was calculated for the investigated field sizes of both energies using the following formula:

(6) $$k_{\mathrm{pol}}=\frac{\left|M_{‐}\right|+\left|M_{+}\right|}{2\left|M_{‐}\right|}$$

where $M_{‐}$ and $M_{+}$ are IC signals for negative and positive bias voltage (300 V for RNC and 200 V for PTW31023) respectively.

RAZOR nanochamber has previously been reported to show a minimal deviation (<0.3%) in recombination factor for small fields^27^ and hence these were not recalculated in this work. However, for PTW31023 these factors need to be investigated for small fields. Two voltage method, as given in Eq. (7), was used to calculate the recombination correction factor (*k_s_*) as it gives linear relation between *1/M* and *1/V* in Jaffe plot^22^ which is the basic condition for using this method.^13^

(7) $$k_{s}=1+\frac{\left(\frac{M_{1}}{M_{2}}\right)‐1}{\left(\frac{V_{1}}{V_{2}}\right)‐1}$$

where $M_{1}$ and $M_{2}$ are the IC’s readings at bias voltage $V_{1}$ (200 V) and $V_{2}$ (100 V) respectively.

Stability time of at least 5 min and preirradiation of 10 Gy was given to each IC following the change of bias voltage on electrometer.

#### 2.B.6 Output ratios (OR)

For OR measurement in small fields, as defined in equation, detector’'s signal (*M*) was corrected for influence quantities like temperature, pressure, polarity, and recombination effects.

(8) $$OR_{f_{\mathrm{clin}},det}=\frac{M_{f_{\mathrm{clin}},det}}{M_{f_{\mathrm{msr}},det}}$$

where $M_{f_{\mathrm{clin}}}$ and $M_{f_{\mathrm{msr}}}$ are corrected average signals of detector for clinical and msr fields respectively.

In order to reduce the uncertainty in measurement, signal for individual small fields was measured interleaving the msr field (10 × 10 cm^2^) measurement as recommended in TRS 483.

#### 2.B.7 Correction factors

Correction factors (CFs) of RD, RNC, and PTW31023 for small field *f_clin_* were calculated using the following formula:

(9) $$CF_{f_{clin,},det}=\frac{FF_{f_{\mathrm{clin}}}}{OR_{f_{\mathrm{clin}},det}}$$

where $FF_{f_{\mathrm{clin}}}$ is the average of the field factors as calculated from different methods defined in Eqs. (2), (3), and (5) whereas $OR_{f_{\mathrm{clin}},det}$ is the output ratio calculated using Eq. (8).

Uncertainty (1 rel. SD) in the readings of each detector was calculated and its propagation for all arithmetic calculations was accounted for determining the final uncertainty in the correction factors.

## 3 RESULTS

### 3.A Effective field size

Table 3 shows the corresponding *S_eff_* (which is the true representative of an irradiation field) for each nominal field defined with jaws and MLCs based on the beam profile employing Eq. (1). It can be seen that the *S_eff_* values are somewhat larger than the nominal field size specifically for 10 MV beam energy and MLC collimated fields (except for MLC collimated reference fields). This increase becomes more significant as the field size is reduced, for instance *S_eff_* of 0.58 cm against nominal field of 0.5 × 0.5 cm^2^, that is, 0.08 mm increase corresponds to 16% increase while *S_eff_* of 4.08 cm against nominal field of 4 × 4 cm^2^ shows an increase of only 2%.

**Table 3:** Effective field sizes (in cm) against different nominal fields.

Nominal field size (cm^2^)		0.5 × 0.5	1 × 1	1.5 × 1.5	2 × 2	2.5 × 2.5	3 × 3	4 × 4	10 × 10
6 MV	Jaws	0.52	1.00	1.53	2.00	2.50	3.00	4.00	10.05
	MLCs	0.56	1.09	1.57	2.08	2.57	3.07	4.05	9.97
10 MV	Jaws	0.54	1.03	1.54	2.00	2.51	3.00	4.00	10.06
	MLCs	0.58	1.11	1.60	2.10	2.60	3.09	4.08	9.98

### 3.B Field factors

**Fig. 3:**
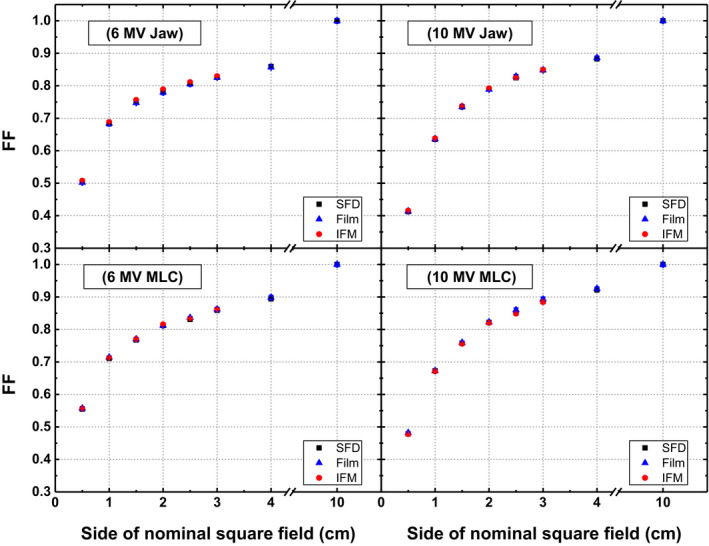
Field factors of jaws (upper panel) and multi leaf collimators (lower panel) collimated fields of 6 MV (left panel) and 10 MV (right panel) photon beams measured with different methods.

Figure 3 shows the comparison between FFs as measured with different methods for 6 and 10 MV jaws and MLCs collimated fields. Difference between the FFs of SFD (for both IFM and direct method) and films has been found to be <1% with maximum difference of 0.92% for 0.5 × 0.5 cm^2^ MLCs defined field of 10 MV beam. FFs measured with SFD (IFM) have been found to be closer to those measured with films for 10 MV jaws fields whereas for 10 MV MLCs fields, SFD (direct method) measured FFs show better agreement with films. The response is mixed for 6 MV fields.

### 3.C Beam profiles and PDDs

**Fig. 4:**
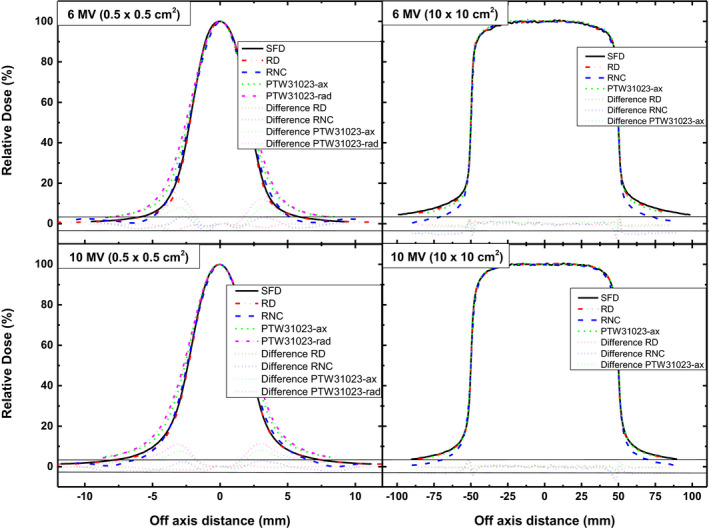
Crossline profiles of 6 MV (upper panel) and 10 MV (lower panel) beams measured with different detectors for 0.5 × 0.5 cm^2^ (left panel) and 10 × 10 cm^2^ (right panel) fields. Difference plots are presented in the bottom. Pair of black solid horizontal lines indicate the limit of ±3%.

**Fig. 5:**
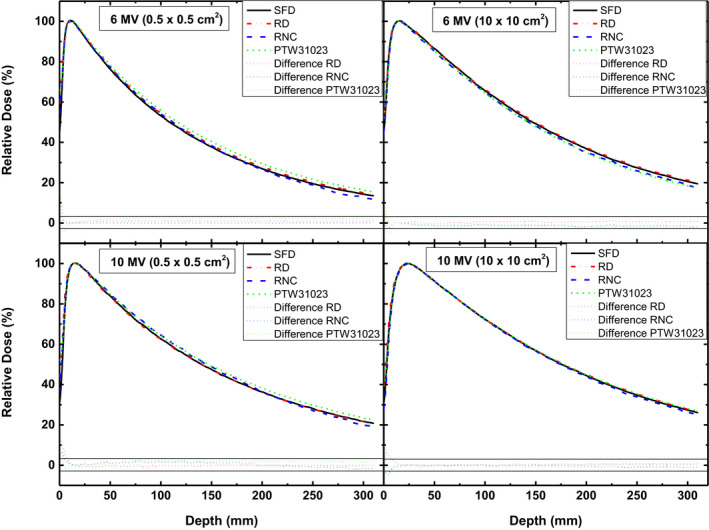
Depth dose distribution of 6 MV (upper panel) and 10 MV (lower panel) beams measured with different detectors for 0.5 × 0.5 cm^2^ (left panel) and 10 × 10 cm^2^ (right panel) fields. Difference plots are presented in the bottom. Pair of black solid horizontal lines indicate the limit of ±3%.

Figures 4 and 5 present the crossline profiles and PDDs acquired for 0.5 × 0.5 cm^2^ and 10 × 10 cm^2^ to highlight the difference in the detectors for measurement of lateral and depth dose distribution in the smallest and largest investigated fields. Difference plots in the bottom of these Figures show the deviation of detectors from SFD measured data. For beam profiles, difference in the response of RD and RNC is up to 3% whereas it exceeds 10% for PTW31023 in 0.5 × 0.5 cm^2^ field of both energies, showing higher difference (up to 15%) in radial orientation. Beam profiles of 10 × 10 cm^2^ show that RNC measured dose is considerably lower in the tail region.

For PDD measurement (Fig. 5), it can be seen that PTW31023 exhibits a maximum deviation of 2.9% and 2.5% for 6 and 10 MV, respectively, beyond build‐up region for smallest field size of 0.5 × 0.5 cm^2^. Whereas in build‐up region this deviation reaches up to 20%. The best agreement between all the detectors is seen for reference field of 10 MV (<1% variation beyond build up region).

Table 4 presents FWHM and penumbra values as measured with several detectors. FWHM measured with SFD, RD, and RNC agrees well with each other with a maximum deviation of 0.2 and 0.3 mm for 6 and 10 MV respectively. While PTW31023 measured values are significantly larger (up to 0.3 and 0.6 mm wider in axial orientation for 6 and 10 MV beams, respectively) as compared to SFD. Penumbra values of both diode detectors are consistent to each other (with maximum difference of 0.2 mm). However, IC measured penumbra is somewhat wider than diodes. For PTW 31023 in axial orientation, its value is up to 0.5 to 0.7 mm larger while for RNC 0.2 to 0.8 mm larger than diode. Radial orientation of PTW31023 widens both the FWHM and penumbra values up to 0.2–0.3 mm (compared to axial direction) as shown for smallest field of 0.5 × 0.5 cm^2^.

**Table 4:** FWHM and Penumbra values of crossline profiles as measured with different detectors. SFD, RD and PTW31023 were placed in axial while RNC was positioned in radial orientation. The values for PTW31023 marked with asterisk (*) are those obtained in radial orientation.

	Nominal field size (cm^2^)	FWHM (mm)				Penumbra (mm)			
		SFD	RD	RNC	PTW31023	SFD	RD	RNC	PTW31023
6 MV	10 × 10	100	100.1	99.9	100.2	4.6	4.4	4.6	4.6
	4 × 4	39.6	39.5	39.4	–	2.8	2.7	3.8	–
	3 × 3	29.6	29.5	29.5	–	2.6	2.4	3.4	–
	2.5 × 2.5	24.6	24.6	24.4	–	2.5	2.4	3.3	–
	2 × 2	19.4	19.5	19.6	19.5	2.3	2.3	3.1	3.0
	1.5 × 1.5	14.8	14.8	14.6	14.6	2.3	2.2	2.8	2.9
	1 × 1	9.3	9.5	9.4	9.6	2.0	2.0	2.4	2.6
	0.5 × 0.5	4.5	4.4	4.6	4.8 5.0*	1.8	1.7	2.0	2.3 2.5*
10 MV	10 × 10	100	99.7	99.9	100.1	5.2	5.0	5.8	5.8
	4 × 4	39.6	39.5	39.4	–	3.9	3.7	4.7	–
	3 × 3	29.4	29.7	29.6	–	3.6	3.4	4.6	–
	2.5 × 2.5	24.6	24.6	24.5	–	3.5	3.3	4.2	–
	2 × 2	19.5	19.6	19.6	19.5	3.2	3.0	4.0	3.8
	1.5 × 1.5	14.9	14.9	14.7	14.7	3.1	3.0	3.4	3.6
	1 × 1	9.5	9.7	9.5	9.8	2.7	2.5	3.0	3.2
	0.5 × 0.5	4.5	4.6	4.8	5.1 5.3*	1.9	2.1	2.2	2.5 2.8*

* Using radial orientation of PTW31023.

### 3.D Polarity and recombination effect of ion chambers

**Fig. 6:**
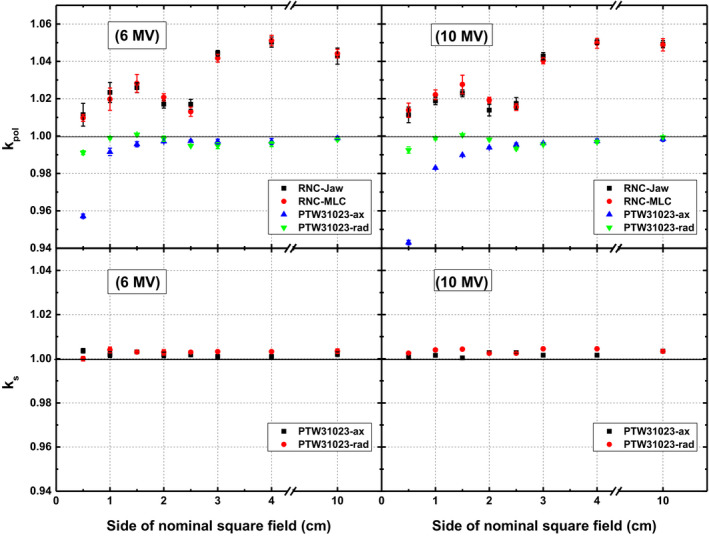
Polarity (upper panel) and recombination (lower panel) correction factors of ICs as a function of field size for 6 MV (left panel) and 10 MV (right panel) beams. Results of PTW31023 are presented for both axial and radial orientation in jaws defined fields.

The polarity (*k_pol_*) and recombination (*k_s_*) correction factors of ICs as a function of field size are plotted for 6 and 10 MV photon beams in Fig. 6. It can be seen that the RNC exhibits a strong polarity effect that varies considerably with field size (in the range of 1.01 to 1.05). However, it has been found practically independent of the collimation system and beam energy in the whole range of field sizes. On the other hand, PTW31023 shows a little variation (<0.4%) in *k_pol_* for field size ≥ 1.0 × 1.0 cm^2^ in radial orientation. Nevertheless, it deviates significantly for smallest fields. A considerable effect of orientation of PTW31023 on polarity effect is also evident in the smaller fields especially for 10 MV beam (Fig. 6 upper panel). Axial placement of PTW31023 gives larger deviation in *k_pol_,* that is, up to 5.9% for 10 MV beam compared to 0.9% deviation in radial direction.

Figure 6 (lower panel) present *k_s_* of PTW31023 for 6 and 10 MV beams. Variation in *k_s_* with field size has been found to be <0.2% for both energies. The overall effect of chamber orientation and energy has been found to be insignificant in all fields.

### 3.E Output ratios and correction factors

**Fig. 7:**
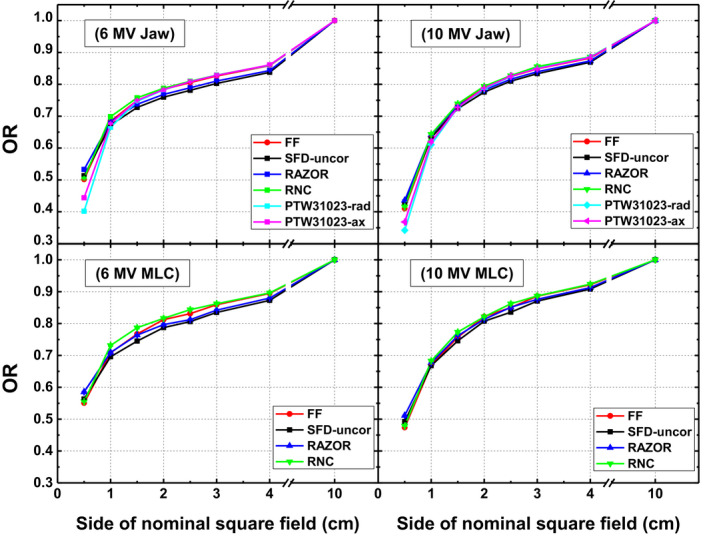
Output ratios of different fields collimated with jaws (upper panel) and multi leaf collimators (lower panel) as measured with several detectors for 6 MV (left panel) and 10 MV (right panel) beams. Field output factors and uncorrected data of stereotactic field diode are also included for comparison.

Figure 7 exhibits ORs as measured with different detectors for jaws and MLCs collimated fields of 6 and 10 MV beam energies. For comparison, FFs (average of films and SFD) are also plotted. ORs measured with RNC are most consistent with FFs, compared to other detectors, which result in CFs closer to unity as shown in Table 5. The maximum difference in OR of RNC and FF has been found to be 2 % for 1 × 1 cm^2^ field of 6 MV MLC beam. RD over responds for fields smaller than 2 × 2 cm^2^ resulting in the ORs larger than FF with maximum deviation of 5.2% for 6 MV jaws field size of 0.5 × 0.5 cm^2^. PTW31023 measured ORs are considerably smaller than FFs for small fields (with maximum deviation of up to 25% for 6 MV 0.5 × 0.5 cm^2^ field). Furthermore, orientation of PTW31023 also affects OR measurement especially for the smallest field size.

**Table 5:** Correction factors of different detectors against nominal field size (effective field size are given in Table 3). Numbers in parenthesis show the uncertainty (1 rel SD).

	Nominal field size (cm^2^)	Razor		Razor NC		PTW31023 (rad)	PTW31023 (ax)
		Jaws	MLCs	Jaws	MLCs	Jaws	
6 MV	10 × 10	1.000 (0.004)	1.000 (0.002)	1.000 (0.004)	1.000 (0.003)	1.000 (0.004)	1.000 (0.004)
	4 × 4	1.018 (0.003)	1.019 (0.004)	0.997 (0.003)	1.000 (0.004)	0.998 (0.004)	0.997 (0.003)
	3 × 3	1.02 (0.002)	1.022 (0.003)	0.998 (0.002)	0.999 (0.003)	0.998 (0.004)	0.999 (0.003)
	2.5 × 2.5	1.023 (0.003)	1.026 (0.002)	0.998 (0.003)	0.988 (0.002)	0.999 (0.004)	0.999 (0.004)
	2 × 2	1.02 (0.004)	1.021 (0.003)	0.996 (0.003)	0.996 (0.002)	1.002 (0.004)	1.001 (0.004)
	1.5 × 1.5	1.019 (0.003)	1.007 (0.003)	0.991 (0.002)	0.981 (0.004)	1.006 (0.003)	1.002 (0.004)
	1 × 1	1.004 (0.004)	1.005 (0.002)	0.981 (0.004)	0.980 (0.004)	1.030 (0.004)	1.012 (0.004)
	0.5 × 0.5	0.948 (0.002)	0.950 (0.003)	0.998 (0.004)	1.002 (0.003)	1.255 (0.003)	1.136 (0.003)
10 MV	10 × 10	1.000 (0.002)	1.000 (0.001)	1.000 (0.002)	1.000 (0.002)	1.000 (0.002)	1.000 (0.002)
	4 × 4	1.012 (0.003)	1.012 (0.003)	0.998 (0.003)	1.000 (0.003)	0.999 (0.003)	1.000 (0.003)
	3 × 3	1.011 (0.001)	1.013 (0.003)	0.992 (0.002)	1.001 (0.003)	0.998 (0.001)	1.000 (0.002)
	2.5 × 2.5	1.013 (0.002)	1.011 (0.003)	0.999 (0.003)	0.998 (0.003)	1.002 (0.002)	1.001 (0.002)
	2 × 2	1.010 (0.002)	1.01 (0.002)	0.995 (0.003)	1.000 (0.002)	1.006 (0.002)	1.002 (0.002)
	1.5 × 1.5	1.004 (0.003)	0.995 (0.002)	0.996 (0.003)	0.980 (0.003)	1.012 (0.002)	1.011 (0.002)
	1 × 1	0.993 (0.002)	0.994 (0.001)	0.989 (0.002)	0.984 (0.002)	1.043 (0.002)	1.027 (0.002)
	0.5 × 0.5	0.950 (0.002)	0.951 (0.001)	0.997 (0.003)	1.005 (0.003)	1.211 (0.002)	1.126 (0.002)

It is evident from Table 5 that the CFs of RNC are ≤2% for all combinations of field size, energy and collimation. PTW31023 has the largest value of CF, that is, up to 1.255 and 1.211 in radial orientation while 1.136 and 1.126 in axial orientation for 0.5 × 0.5 cm^2^ field of 6 and 10 MV beams respectively). CFs of RD are well <2% for field sizes down to 1 × 1 cm^2^ of both energies while for smallest field its value increases to 5.2% (i.e. 0.948). Variation in CFs of all detectors has been found to be independent of the collimation system (with variation of <1.1% between MLCs and Jaws defined fields) but dependent on the effective field size.

## 4 DISCUSSION

In this work, it is has been found that the effective field sizes of both energies are significantly different from nominal fields particularly for smallest field size (Table 3). Therefore, these are required to be measured for accurate determination of CFs.

For FFs, a good agreement (<1% variation) has been found between EBT3 films and SFD measurements (employing both direct and IFM methods) for all combinations of field size, energy, and collimation system (Fig. 3). This infers that SFD with corresponding CFs provided in TRS 483 may be used to determine the FFs accurately for both energies. Furthermore, the linear interpolation technique may be used reliably to calculate CFs, and hence corresponding FFs, for those effective fields that are not provided in TRS 483.This finding supports using SFD as a reference detector effectively to find CFs of new detectors (using detector‐to‐detector correction approach) where gold standard EBT films are not available. Regarding the accuracy, both IFM and direct methods have been found equally consistent with films in this work. Although, Dieterich et al.^34^ have shown the superiority of IFM over direct method explaining that IFM minimizes the error in FF measurement with high density silicon detector like SFD by mitigating its stronger field size dependence in the range of intermediate to reference field. On the contrary, Denia et al.^32^ have found that the direct method is more accurate as IFM increases the uncertainty by depending on electrometer readings more.

Difference in the beam profiles of smallest field measured with different detectors as shown in Fig. 4 may be attributed mainly to the variation in their active volume. Negligible difference in the response of RD and SFD is due to the same radial dimensions (diameter of 0.6 mm) of their active material. Largest difference exhibited by PTW 31023 (especially in radial orientation)is ascribed to its large size and hence is not suitable for profiling in small fields. Unlike other reported ICs, RNC agrees well with high resolution diode detectors with a maximum difference of 3% for smallest field size of 6 MV beam which makes it reasonably good for profile measurements. However, the crossline profiles measured with RNC show a much lower dose in the tail region (Fig. 4 right panel). This phenomenon is unusual and may be associated to the considerably high uncertainty and lower signal to noise ratio of small volume RNC in the low dose region.

For PDD measurement, response of all detectors used in this study agree well with each other (<3% variation)beyond build up region (Fig. 5). Generally, all detectors used in this study show better consistence for 10 MV beam profile and PDD measurement as compared to 6 MV beam. Over response of unshielded diode detectors is negligible for fields up to 10 × 10 cm^2^, which may become considerable in larger fields where scattered component is high and this finding is consistent with reported literature.^35^

It is worth mentioning that the suitability of SFD, used as a reference detector in this work, for profiling and agreement with high resolution radiochromic films is frequently reported.^36^, ^37^ Additionally, TRS 483 recommends using unshielded diode for beam profiles measurement in small fields. Variation in the overresponse of SFD as a function of depth is also very minimal and hence can be used as a reference detector for measuring PDD (beyond build up region) for field size as large as 10 × 10 cm^2^.^36^, ^38^, ^39^

Regarding ICs, RNC shows a strong polarity effect compared to PTW31023, as obvious from Fig. 6, which may be ascribed to its small size.^25^ Spherical chamber design of RNC may also be a factor causing the larger polarity effect that may be reduced by increasing the length with smaller diameter of sensitive volume of IC (as in cylindrical shape of PTW31023).^40^ Variation in *k_pol_* of RNC with field size is significantly high, that is, up to 4% while it is independent of energy and collimation system. On contrary, for PTW31023 it varies only <0.4% in radial orientation for fields ≥1.0 × 1.0 cm^2^ of both energies. Greater variation in polarity effect of PTW31023 as observed in axial direction may be attributed to the cable irradiation and stem effect that is consistent with the literature reported for PTW31014.^25^, ^40^ Furthermore, deviation in *k_s_* of PTW31023 in both orientations (axial and radial) is insignificant (<0.2%) for all field sizes of both 6 and 10 MV beam energies. These specifications make PTW31023 suitable to be used as reference class IC in above‐mentioned conditions.^13^, ^41^

For OR measurement, overresponse of diode detectors in small fields is attributed to the fluence perturbation caused by high density silicon component in the active volume of detector and is consistent with other unshielded silicon detectors.^17^, ^21^, ^42^ Considering the limit of usability of a detector to be 2% as recommended in literature,^15^, ^16^, ^17^ CFs of RD are observed in the range of up to 2% for both energies except for the smallest field of 0.5 × 0.5 cm^2^ where it increases to 5.2%.

ORs of pinpoint chamber PTW31023 are smaller than field factors due to the volume effect rendered by its finite size (0.015 cc). Radial orientation of this cylindrical shaped IC exhibits larger volume averaging effect and hence higher value of CF compared to axial direction. Range of ±2% of CF for PTW31023 is limited to the field size down to 1.5 × 1.5 cm^2^.While it cannot be used for FF measurement in smallest field (0.5 × 0.5 cm^2^) in any orientation for which its CF value is tremendously higher (up to 25.5% and 13.6% in radial and axial orientations respectively) than TRS 483 recommended limit (i.e. ±5%).

Response of RNC is interesting in small fields. Probably two counteracting phenomena are occurring simultaneously; one being the under response of detector due to its finite size, while the other is the over response due to fluence perturbation caused by the presence of high density components like graphite (1.81 g/cm^3^) electrode and shonka (1.76 g/cm^3^) wall. Due to its extremely small size (0.003 cc), volume effect of RNC is substantially small and probably would be suppressed by the overresponse due to the presence of high density materials and therefore result in correction factors smaller than unity unlike all other small field air chambers. The same trend of over response has been observed by Looe et al.^25^

**Fig. 8:**
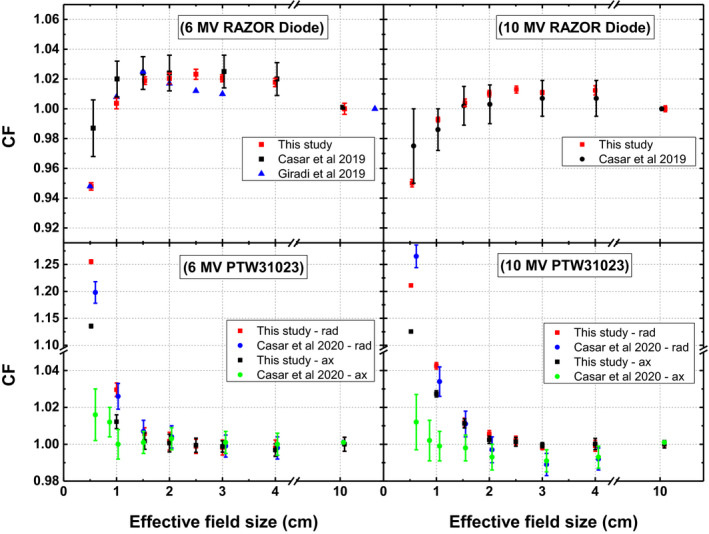
Comparison of correction factors calculated in this study with published data. RAZOR diode (RD) (upper panel) and PTW31023 (lower panel) data is presented for 6 MV (left panel) and 10 MV (right panel) beam. RD data of Giradi et al presented in left upper panel has been published for nominal field size with msr of 10.4 × 10.4 cm^2^.

Comparison of CFs calculated in this work and those reported in literature are presented in Fig. 8. RD follows the same trend as reported by Casar et al and Giradi et al.^42^, ^43^ For 6 MV beam, CFs calculated in this study are in a better agreement with those calculated by Giradi et al^43^ exhibiting a deviation of <1%. Whereas Casar et al^42^ determined CFs exhibit a difference of up to 4% for 0.5 × 0.5 cm^2^ field, although for 10 MV beam this difference decreases to approximately 2.5 %. For PTW31023, the difference in CF measured in this study and that of Casar et al^24^ is <1.5% for fields smaller than 1 × 1 cm^2^ for both energies. Whereas a substantial deviation has been observed for smaller fields. This difference becomes significantly larger (up to 10%) for smallest field size in axial orientation (Figure:lower panel) and may be attributed to considerably larger polarity effect of PTW31023 in this orientation that is not accounted for in Casar et al’s study.

## 5 CONCLUSIONS

Following conclusions are drawn in this work.

- SFD (with corresponding CFs provided TRS 483) may be used as substitute of EBT3 films to determine the FFs of small fields accurately. Linear interpolation method may be employed reliably to calculate the CFs of those intermediate effective fields for which the data are not available in TRS 483.
- RD and RNC may be used for measuring beam profiles and PDDs (beyond build up region) in small fields with an accuracy of up to 3%. PTW31023 is not an appropriate detector for beam profiling though it may be used for PDD measurement.
- PTW31023 may be used as reference class IC in radial direction for fields ≥ 1 × 1 cm^2^ as its *k_pol_* and *k_s_* values are within limits described in TRS 483 for these fields. Whereas RNC has considerably higher value of *k_pol_* that shows a significant variation with field size.
- CFs of a detector does not depend on collimation system but dependent on the effective field size and energy. In axial orientation, PTW31023 shows considerably smaller CF compared to radial direction.
- RNC may be used in all fields investigated in this study without any CF considering limit of usability of a detector to be 2%. RD and PTW31023 may be used without CF for fields down to 1 × 1 cm^2^ and 1.5 × 1.5 cm^2^ respectively. While smaller fields need to get appropriate CFs. However, to achieve a higher level of accuracy (>2%), corrections are required for each detector in all field sizes.

## CONFLICT OF INTEREST

There is no conflict of interest.
